# Development and application of the Meal and Snack Assessment (MESA) quality scale for children and adolescents using item response theory

**DOI:** 10.1186/s12937-024-00948-y

**Published:** 2024-05-14

**Authors:** Stella Lemke, Dalton Francisco de Andrade, Patrícia de Fragas Hinnig, Silvio Aparecido da Silva, Silvana Ligia Vincenzi, Denise Miguel Teixeira Roberto, Adriana Soares Lobo, Francilene Gracieli Kunradi Vieira, Patricia Faria Di Pietro, Maria Alice Altenburg de Assis

**Affiliations:** 1 Nutrition Postgraduate Program, Department of Nutrition, Health Sciences Center, Federal University of Santa Catarina, Florianópolis, Brazil; 2 Informatics and Statistics Department, Technological Center, Federal University of Santa Catarina, Florianópolis, Brazil; 3 Knowledge Management Department, Federal Institute of Education, Science and Technology of Santa Catarina, Florianópolis, Brazil; 4 Federal University of Santa Catarina, Campus Trindade, Florianópolis, Santa Catarina 88040-370 Brazil

**Keywords:** Scales, Diet quality, Mealtimes, Ultra-processed foods

## Abstract

**Background:**

Meals differ in terms of food items and nutritional quality. The aim of the present study was to propose a scale to measure the meals quality of schoolchildren according to food processing degree, perform a preliminary evaluation of the scale's validity and reliability and apply the scale to a representative sample of schoolchildren in a city in southern Brazil.

**Methods:**

A methodological study based on the generalized graded unfolding model (GGUM) of item response theory (IRT) with analysis of secondary data was carried out in 6,399 schoolchildren of 6-15y-old attending 2nd to 5th grades of public elementary schools in Florianópolis, Brazil, in 2013–2015 who answered the validated Food Intake and Physical Activities of Schoolchildren (WebCAAFE) questionnaire. Meal quality was the latent trait. The steps for the development of the scale included: latent trait definition; item generation; dimensionality analysis; estimation of item parameters; scale levels definition; assessment of validity and reliability; and assessment of the meal quality of a subsample of schoolchildren aged 7 to 12 years (*n* = 6,372).

**Results:**

Eleven out of eighteen items had adequate parameters, without differential item functioning for sex or age. Meal quality was categorized into three levels: healthy, mixed, and unhealthy. Higher scores indicate a greater prevalence of ultra-processed foods in daily meals. Most schoolchildren had mixed (40.6%) and unhealthy (41%) meal patterns.

**Conclusions:**

IRT analysis allowed the development of the scale, which measures the quality of meals and snacks based on the degree of food processing. At all snack times, there was a higher frequency of ultra-processed foods consumption, therefore foods consumed as snacks are a potential focus for nutritional interventions.

**Supplementary Information:**

The online version contains supplementary material available at 10.1186/s12937-024-00948-y.

## Background

Assessment of dietary intake by eating occasions through empirical derivation of meal patterns [1, 2] and the development of indices to evaluate meal quality [3–5] is an emerging field of research. This approach allows understanding how different meal patterns can influence diet quality and health outcomes, emphasizing particularities that would not be revealed by overall dietary analyses [6]. A systematic review of indices developed to assess the nutritional quality of meals identified seven studies, most of which were carried out to evaluate a single meal [3]. Of the seven studies, two developed tools to assess breakfast quality in children, adolescents, and young adults [7, 8]; three analyzed lunch quality in students and workers [9–11]; one evaluated dinner quality in adults [12]; and one evaluated all types of meals and snacks in elderly women [13]. The main components for assessing meal quality included total fat or some specific type of fat, fruits, vegetables, cereals, and whole grains. None of them analyzed food consumption according to the degree of food processing [3]. Recently, the Main Meal Quality Index [4] was developed to evaluate the quality of the main meal (usually lunch), and the Meal Balance Index [5] was developed to assess the nutritional quality of meals reported by the North American population. All instruments were developed using the classical test theory (CTT), which is sample-and test-dependent. This means that measure’s properties under CTT may vary across different populations: subject's ability will be dependent of the particular choice of items that are administered, and item statistics will be dependent on the particular choice of examinee sample [14]. It was not possible to identify methodological studies that developed indicators to evaluate the nutritional quality of meals based on item response theory (IRT). Previous studies used IRT to develop scales to evaluate diet quality in children [15] and adults [16], but focused on overall diet quality. IRT is a psychometric method that provides probabilistic model-based measurements, used to develop and refine measures, representing a new approach in nutrition research. It expresses the probability of responding to an item as a function of individual characteristics (latent traits) and item parameters. A fundamental application of IRT lies in the evaluation and improvement of the metric properties of items, aiding in the selection of items that better discriminate the latent trait. Furthermore, IRT allows respondents and items to be placed on the same scale, thus providing an interpretation of the measure in terms of the latent trait being studied [14]. This is the first study to develop a scale to assess six daily meals using probabilistic IRT modeling. Meal quality was treated as a latent trait and defined according to food consumption behavior and the degree of food processing [17], under the premise that the healthier the meal, the greater the predominance of unprocessed/minimally processed foods over ultra-processed foods [18, 19]. This study contributes to advancing the current body of knowledge by developing novel approaches to define and analyze dietary quality, by a single indicator to assess the quality of six daily meals. This study aims (i) to develop a scale to assess the quality of daily meals of schoolchildren according to food processing degree, (ii) to perform a preliminary evaluation of the scale's validity and reliability, and (iii) to measure the quality of meals and snacks of children and adolescents by applying the scale to a representative sample of schoolchildren in a city in southern Brazil.

## Methods

**Fig. 1:**
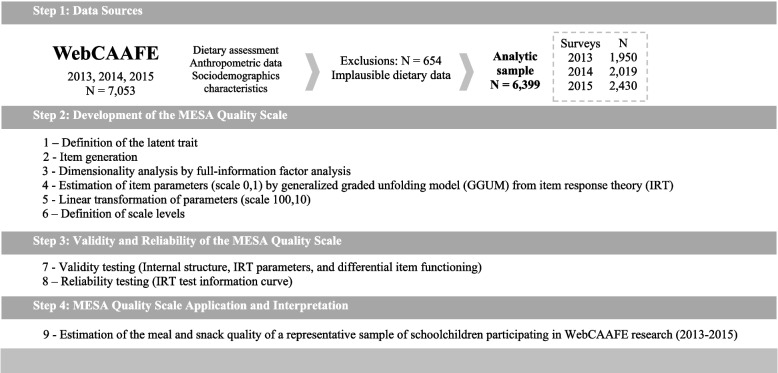
Study design flowchart. The Meal and Snack Assessment (MESA) Quality Scale development was guided by recommendations for psychometric studies, a science that studies the measurement of non-directly observable phenomena (latent traits): Latent trait definition (1); Item generation (2); Dimensionality analysis (3); Estimation of item parameters (4); Linear transformation of parameters (5); Scale levels definition (6); Assessment of scale's validity (7), and reliability (8); Estimation of the latent trait of the individuals in the sample (9)

This methodological study based on IRT used data collected with the Food Intake and Physical Activity of Schoolchildren (WebCAAFE) questionnaire to develop and validate the Meal and Snack Assessment (MESA) quality scale. The developed scale was tested on a representative sample of schoolchildren. A flowchart of the study design is presented in Fig. 1.

### Step 1: Data source

#### Study population and sampling design

The description of the design and sampling procedure has been published previously [20]. In brief, data collection was conducted from August to October 2013, 2014, and 2015 in Florianópolis, Santa Catarina, Brazil. The target population comprised schoolchildren of 6–15-y-old attending from the 2nd to 5th grade of municipal elementary schools equipped with computer rooms and internet access. The primary sampling units were eligible classrooms randomly selected from a complete list of schools with computer rooms provided by the education authority (34 out of 37 schools with 6,227 students enrolled in 2013; 34 out of 36 schools with 6,500 students in 2014; 35 out of 36 schools with 7,104 students in 2015). School years were considered secondary sampling units, with four classes from each educational unit being randomly selected, one from each year. All students from the selected classes were invited to participate in the study. Surveys included a total sample of 7,053 schoolchildren (2013–2015). Of these, 654 children with implausible dietary data were excluded (222 for reporting consumption of fewer than four food items per day and 432 for reporting values three times greater than the standard deviation of the mean). The final sample for the scale development included 6,399 students (1,950 in 2013, 2,019 in 2014, and 2,430 in 2015). For the IRT analyses, samples of around 250–500 respondents with different levels of the latent trace are often recommended [21].

#### WebCAAFE

WebCAAFE is an online questionnaire developed for the periodic assessment of dietary intake, physical activity, and sedentary behavior in students from the 2nd to the 5th grade of public schools. It collects data on the previous day (24-h recall) and comprises three sections, namely general information, dietary intake, and physical activity/sedentary behaviors [22]. WebCAAFE was tested for both reproducibility and validity [23–26]. A demonstrative version is available at https://caafe.ufsc.br. The dietary intake section includes six eating occasions (breakfast, morning snack, lunch, afternoon snack, dinner, and evening snack), each illustrated with up to 32 food icons (rice, vegetables, greens leaves, vegetable soup, beans, cassava flour, corn/potatoes/mashed potatoes, pasta, instant pasta, French fries, beef/poultry, sausages, eggs, fish/seafood, fruits, bread, cheese bread, cream cookies, breakfast cereal, porridge, cheese, coffee with milk, milk, yogurt, chocolate milk, fruit juices, sodas, sweets, packaged snacks, pizza/hamburger/hot dog, nuggets, and plain cake). This section was used to develop the MESA. Schoolchildren selected the foods consumed at each meal of the previous day. The instrument does not allow identifying food amounts or portions and, therefore, does not provide information on total energy or nutrient intake. The objective is to investigate the consumption of healthy and unhealthy foods. Additionally, schoolchildren answer questions about school meals, including the frequency of school meal consumption (0–1, 2–3, or 4–5 times/week). The physical activity and sedentary behaviors were described in detail by Lobo et al. (2019) [20] and assessed by three periods of the day (morning, afternoon, and night). The subject's physical activity score (PAS) was the sum of all activity scores. The variable was categorized into tertiles. The daily frequency of screen-based sedentary activities (television, videogame, computer, tablet, cell phone) was determined for each child and categorized into tertiles. These data were used to assess the quality of schoolchildren's meals in terms of the population description. The instrument was applied once to each child, in computer rooms at schools, in the presence of trained researchers. The day on which the questionnaire was applied differed among children.

#### Anthropometric measurements

Weight and height were measured by trained anthropometrists using standardized procedures [27]. The schoolchildren were barefoot and wearing lightweight clothing. Weight was measured with a digital scale (Marte, model PP, 180 kg maximum capacity, 50 g precision, São Paulo, Brazil). Height was measured using a portable stadiometer (AlturExata®, 1 mm precision, Belo Horizonte, Brazil). The body mass index (BMI) was computed as weight (kg) divided by height squared (m^2^). Age- and sex-specific BMI Z-scores were calculated according to the World Health Organization [28]. Weight status was categorized as non-overweight (thinness and normal weight, BMI Z-score for age <  + 1) or overweight including obesity (BMI Z-score for age ≥  + 1). Anthropometric data were collected on the same day that the schoolchildren answered the WebCAAFE questionnaire.

### Other variables

Information on sex, date of birth, and school shift was provided by the administrative sector of the schools. Age was calculated using the child's date of birth and the date of data collection and categorized as 7–9 and 10–12 years. Family income was estimated from the average income of the census tract of the school′s location available from the Brazilian Institute of Geography and Statistics (IBGE) [29]. The variable was categorized into tertiles.

### Step 2: Development of the MESA scale

#### Latent trait

Although diet quality is a multidimensional concept that encapsulates, among others, nutritional quality (food diversity, dietary adequacy, nutrient density), sensory organoleptic quality, food safety, the social dimension of food [30]. For this study, meal quality was defined considering the current recommendations [18, 19] for choosing and combining foods to compose a healthy meal. Healthy meals are based on a great variety of unprocessed or minimally processed foods, balanced across food groups, while restricting ultra-processed foods [18, 19]. In a healthy diet, processed foods may be consumed in small quantities as ingredients in culinary preparations or as part of meals based on unprocessed/minimally processed foods [11].

#### Item generation

WebCAAFE foods were classified according to the NOVA [17] system into three groups, as follows: (i) unprocessed/minimally processed foods (MPF), including rice, green leaves, vegetables, vegetable soup, beans, cassava flour, corn/potatoes/mashed potatoes, pasta, beef/poultry, eggs, fish/seafood, fruit, porridge, coffee with milk, milk, plain cake; (ii) processed foods (PF), including bread and cheese; and (iii) ultra-processed foods (UPF), including instant pasta, French fries, sausages, cheese bread, cream cookies, breakfast cereal, yogurt, chocolate milk, fruit juices, sodas, sweets, chips, pizza/hamburger/hot dogs, nuggets. The group of processed culinary ingredients was not included because none of the WebCAAFE foods are classified as such. For the IRT analyses, three items (consumption of MPF, PF, and UPF) were proposed to represent the foods on each of the six eating occasions, totaling 18 items. Two response categories were defined: non-consumption and consumption. WebCAAFE consumption reports were used to determine the consumption or non-consumption of MPF, PF, and UPF by each schoolchild in each of the six daily meals. A child was classified as having consumed MPF, PF, or UPF in the meal if they had consumed at least one food from the WebCAAFE classified as MPF, PF, or UPF.

#### Dimensionality

Dimensionality was evaluated by full-information factor analysis with oblimin rotation. The set of items was considered unidimensional when a dominant factor explained more than 20% of the data variation [31]. Factor loadings (≥ 0.3) and commonality (≥ 0.2) were also considered.

#### Item parameters

Discrimination (α) and location (δ) parameters were estimated using the generalized graded unfolding model (GGUM) [32] of IRT, represented by the following equation:

$${\text{P}}\left({\text{Z}}={{\text{z}}1\uptheta }_{{\text{j}}}\right)=\frac{{\text{exp}}\left[{{\text{a}}}_{{\text{i}}}\left({\text{z}}\left({\uptheta }_{{\text{j}}}-{\updelta }_{{\text{i}}}\right)-\sum\limits_{{\text{k}}=0}^{z}{\uptau }_{{\text{ik}}}\right)\right]+{\text{exp}}\left[{{\text{a}}}_{{\text{i}}}\left(\left({\text{M}}-{\text{z}}\right)\left({\uptheta }_{{\text{j}}}-{\updelta }_{{\text{i}}}\right)-\sum\limits_{{\text{k}}=0}^{{\text{z}}}{\uptau }_{{\text{ik}}}\right)\right]}{\sum\limits_{{\text{v}}=0}^{{\text{H}}}\left[{\text{exp}}\left({{\text{a}}}_{{\text{i}}}\left[{\text{v}}\left({\uptheta }_{{\text{j}}}-{\updelta }_{{\text{i}}}\right)-\sum\limits_{{\text{k}}=0}^{{\text{v}}}{\uptau }_{{\text{ik}}}\right]\right)+{\text{exp}}\left({{\text{a}}}_{{\text{i}}}\left[\left({\text{M}}-{\text{v}}\right)\left({\uptheta }_{{\text{j}}}-{\updelta }_{{\text{i}}}\right)-\sum\limits_{{\text{k}}=0}^{{\text{v}}}{\tau }_{{\text{ik}}}\right]\right)\right]}$$

where:

- *Zi* = observable response to item i;
- *z* = 0, 1, 2, 3, ..., *H*; with *z* = 0 representing the strongest level of disagreement and *z* = H representing the strongest level of agreement;
- *θj* = parameter of location of individual j on the latent trait scale, also called individual score or individual latent trait;
- *αi* = discrimination parameter of item i;
- *δi* = location parameter of item i on the latent trait scale;
- *τik* = threshold location parameter of subjective response category k on the latent trait scale relative to the position of item i;
- *H* = number of observable response categories minus 1; and
- *M* = 2*H* + 1.

The equation quantifies the probability of an item response as a function of latent trait and item parameters, represented by the item characteristic curve (ICC). Unfolding models are proximity models developed for attitude, behavior, and preference measures. Individuals and items are expressed in the same metric along the latent trait continuum. The probability of a positive response increases as the individual's value in the latent trait is close to the item's value. Individuals with a latent trait level close to the item will be more likely to agree with the item. IRT uses the individual's response to items and the psychometric property of items themselves to generate a score that represents the latent trait measure [32]. For computational convenience and based on the principle of invariance, all item parameters were initially represented on a (0,1) scale, with 0 representing the mean and 1 the standard deviation of the respondents [21]. The discrimination parameter (α) indicates the ability to discriminate individuals with different latent trait levels, serving as a measure of item quality. Items with α ≥ 0.7, on the (0,1) scale, provide better discrimination of the latent trait. The location parameter (δ) identifies the item's position on the latent trait continuum [33]. Values are expected to be in the range of –2 to + 2 [34]. Item parameters were estimated by the maximum marginal likelihood method and analyzed in terms of standard error (SE) and ICC. Estimates of individual parameters (scores) were obtained by the Bayesian expected a posteriori method [32]. Analyses were performed using the MIRT package in software RStudio v.1.2.5033 (RStudio Team, 2020).

#### Linear transformation of parameters

To facilitate the development and interpretation of the scale, avoiding negative or decimal numbers, we performed the linear transformation of parameters to mean 100 and standard deviation 10 (scale 100,10).

#### Definition of scale levels

The probability of consumption and non-consumption of each item in the six daily meals, calculated using ICC, is represented on the scale. The cut-off points for each level considered the gain in diet quality with food intake characteristics (MPF, UPF, or both). Three levels of meal quality were established, namely healthy, mixed, and unhealthy. This step was performed by an expert. Scale levels and their descriptions were reviewed by authors.

### Step 3: Validity and reliability of the MESA scale

Evidence of construct validity was obtained by the analysis of the internal structure, estimation of IRT parameters, and differential item functioning (DIF) [35] for sex and age. DIF is present when an item behaves differently between two or more groups of individuals with the same level of a latent trait. A lack of DIF is indicative of good construct validity [21]. The assessment of scale reliability was performed using the IRT test information curve (TIC).

### Step 4: MESA Application and interpretation

The scale was applied to a subsample of elementary schoolchildren from Florianópolis, Brazil (*n* = 6,372), to estimate the quality of their meals using the sampling plan created for data collection through the WebCAAFE surveys from 2013 to 2015. The sampling plan was stratified into two levels, the strata being the combination of year and school; level 1 was the class, and level 2 was the student within the class. For interpretation, items and individuals were simultaneously positioned on the latent trait continuum, with the individual's parameter being allocated according to their score, and items being allocated according to the values of their location parameters. Students with scores close to the item's position probably agree with the item. The 95% confidence interval (95% CI) was used to analyze differences between survey years and meal quality levels as well as between meal quality levels and the other variables. Analyses were performed using STATA 16.1 (StataCorp,2020). All analyses considered the effect of study design, incorporating sample weights through the “svy” command in STATA.

## Results

### Development of the MESA scale

The assumption of unidimensionality was supported by the full-information factorial analysis, which showed the presence of a dominant factor explaining 27.4% of the total variance.

Items related to MPF and UPF consumption at breakfast; MPF, PF, and UPF at morning snack time; UPF at lunch; MPF and UPF at afternoon snack time; UPF at dinner; and MPF and UPF at evening snack time had adequate values of factor loading (≥ 0.3) and/or commonality (≥ 0.2) and discrimination (≥ 0.7) and were thus retained in the final scale (Table 1). Seven items did not fit the model well and were not included, namely items related to PF consumption at breakfast, lunch, afternoon snack, dinner, and evening snack; and MPF consumption at lunch and dinner.

**Table 1:** Factor loadings, communalities and parameter estimates of MESA scale items

Item	Factor loadings	Commonality	α (SE)	δ (SE)
(I01) MPF consumption at breakfast	0.54	0.30	1.10 (0.14)	-0.88 (0.11)
(I10) MPF consumption at afternoon snack	0.50	0.25	0.98 (0.11)	-0.72 (0.10)
(I16) MPF consumption at evening snack	0.52	0.27	1.04 (0.12)	-0.55 (0.08)
(I04) MPF consumption at morning snack	0.55	0.30	1.11 (0.12)	-0.47 (0.08)
(I05) PF consumption at morning snack	0.38	0.14	0.70 (0.12)	-0.17 (0.13)
(I06) UPF consumption at morning snack	0.63	0.39	1.37 (0.14)	0.14 (0.06)
(I18) UPF consumption at evening snack	0.55	0.31	1.13 (0.10)	0.36 (0.07)
(I09) UPF consumption at lunch	0.56	0.31	1.14 (0.10)	0.38 (0.08)
(I15) UPF consumption at dinner	0.54	0.29	1.08 (0.10)	0.42 (0.08)
(I12) UPF consumption at afternoon snack	0.45	0.20	0.85 (0.08)	0.62 (0.11)
(I03) UPF consumption at breakfast	0.51	0.26	1.01 (0.09)	0.75 (0.11)

*(α)* discrimination parameter of item, *(δ)* location of item, *SE* standard errors, *MESA* Meal and Snack Assessment Quality Scale, *MPF* unprocessed/minimally processed foods, *PF* processed foods, *UPF* ultra-processed foods

Discrimination parameters ranged from 0.70 to 1.37. Consumption of PF at morning snack time had the lowest discrimination value, indicating that it contributed the least to differentiating schoolchildren with respect to the latent trait. UPF consumption at morning snack time had the highest discrimination value.

Location parameters extended in both negative and positive directions, ranging from –0.88 to 0.75, demonstrating the existence of items that can measure the entire latent trait continuum. Items were logically ordered in ascending order of location parameter value, as follow: items related to MPF consumption, with a negative value; item related to PF consumption, with a more neutral value; and items related to UPF consumption, with a positive value.

**Fig. 2:**
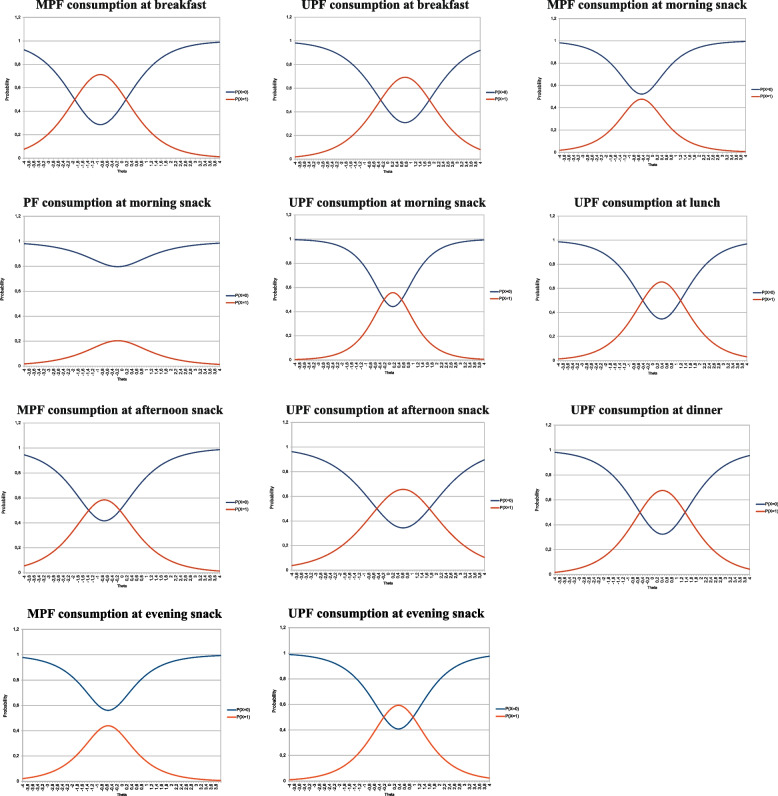
Items Characteristic Curve (ICC) (scale 0,1). The ordinate axis represents the probability of non-consumption (P(X = 0)) or consumption (P(X = 1)) and the abscissa axis represents the individual score or individual latent trait (Theta). The ICC describes how a change in latent trait level is related to a change in item response probability. Abbreviations: MPF = unprocessed/minimally processed foods; PF = processed foods; UPF = ultra-processed foods

The ICC of items is presented in Fig. 2. The ICC can be interpreted in the following manner. Regarding, for instance, item related to MPF consumption at breakfast, schoolchildren with meal quality scores between –1.9 and 0.1 are more likely to consume MPF at breakfast. Schoolchildren with meal quality scores below –1.9 or above 0.1 are more likely not to consume MPF at breakfast. It should be noted that the ICC of the item related to MPF consumption at morning snack time has a different characteristic: the probability curves of the response categories do not intersect, demonstrating that, regardless of the child's latent trait, the probability of not consuming MPF at the morning snack time is greater than that of consuming it. The same applies to items related to PF consumption at morning snack time and MPF consumption at evening snack time.

**Fig. 3:**
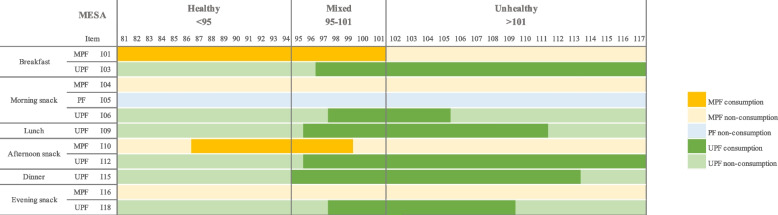
Meal and Snack Assessment (MESA) Quality Scale (scale 100,10). Level 1: Healthy (score < 95), the meal pattern is based on MPF consumption; there is a greater probability of consuming MPF at breakfast and afternoon snack time and not consuming UPF at other daily meals. Level 2: Mixed (95 ≤ score ≤ 101), the meal pattern is based on MPF and UPF consumption; there is a greater probability of consuming MPF at breakfast and afternoon snack time and of consuming UPF at breakfast, morning snack, lunch, afternoon snack, dinner, and evening snack time. Individuals with medium scores have both healthy and unhealthy eating practices. Level 3: Unhealthy (score > 101), the meal pattern is based on UPF consumption; there is a greater probability of consuming UPF and not consuming MPF at all daily meals. Abbreviations: MPF = unprocessed/minimally processed foods; PF = processed foods; UPF = ultra-processed foods

MESA results are shown in Fig. 3. Three levels were determined, namely healthy (score < 95), mixed (95 ≤ score ≤ 101), and unhealthy (score > 101). It is concluded that the lower the score, the greater the presence of MPF and the lower the presence of UPF at daily meals, indicating higher-quality meals.

### MESA Validity and reliability

#### DIF

No item had differential behavior, indicating that the scale measures the quality of the schoolchildren's meals with the same precision, regardless of sex or age (see Additional file 1).

#### TIC

**Fig. 4:**
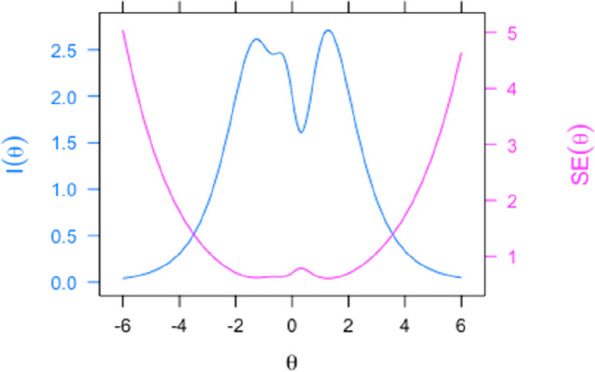
Test Information (I) and standard error (SE) curves of the MESA Quality Scale (scale 0,1). The graph indicates the region of the scale where the measurement of meal quality is most accurate. The higher the information value and the lower the standard error of measurement, the greater the accuracy of the meal quality estimates

Figure 4 shows the TIC, which represents the information sum of all items on the scale. The instrument was shown to cover the entire latent trait, with better accuracy from –1.8 to 1.8 (scale 0,1), corresponding to scores of 82 to 118 (scale 100,10).

### MESA Application and interpretation

#### Meal quality of schoolchildren

**Fig. 5:**
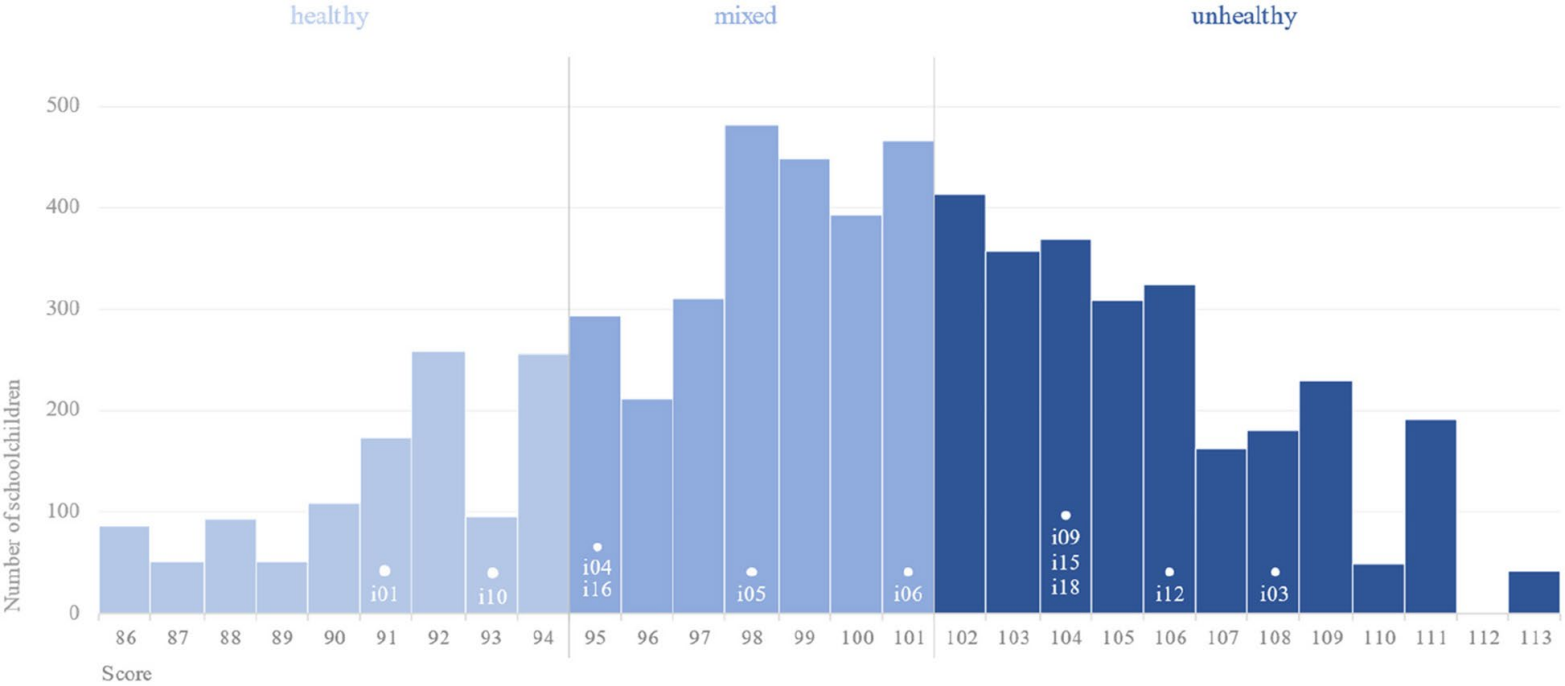
Item location and distribution of schoolchildren by score (scale 100,10). The figure shows the location of the items and the distribution of schoolchildren by score placed on the same scale. Schoolchildren with scores close to the location of the item will be more likely to agree with the item (give a positive response). The items are related to MPF (i01) and UPF (i03) consumption at breakfast; MPF (i04), PF (i05), and UPF (i06) consumption at morning snack time; UPF (i09) consumption at lunch; MPF (i10) and UPF (i12) consumption at afternoon snack time; UPF (i15) consumption at dinner; and MPF (i16) and UPF consumption (i18) at evening snack time. Abbreviations: MPF = unprocessed/minimally processed foods; PF = processed foods; UPF = ultra-processed foods

Students' latent traits ranged from 86 to 113 (scale 100,10). Figure 5 shows the item location and distribution of schoolchildren by score. We highlight the concentration of schoolchildren near the item related to UPF consumption at morning snack time. Yogurt, cream cookies, chocolate milk, and fruit juices were the most reported UPF consumed as morning snack.

#### Population characteristics

Of the total sample, 50% were boys and 61% were aged 7–9 years. The prevalence of overweight was 35%. Additionally, 75% of dietary reports referred to the weekday, and 53% reported consuming school meals 4–5 times/week.

The proportion and 95% CI of schoolchildren at each scale level, based on the three years of WebCAAFE research (2013–2015), are presented in Table 2. Most schoolchildren were classified as having mixed (40.6%) and unhealthy (41%) meal patterns in the three years of the study. The proportion of schoolchildren at each level did not differ according to survey years, school shift, weight status, or day of food intake report. A greater proportion of schoolchildren in the 1st and 2nd tertile of PAS and screen activity (as opposed to the 3rd tertile) was classified as healthy. A greater proportion of schoolchildren with a higher frequency of school meal consumption (4–5 times/week vs. 0–1 times/week) and higher PAS were found to have a mixed meal quality pattern. Boys, those aged 10–12 years, those classified in the 3rd tertile of family income (as opposed to the 1st tertile), and those who reported consuming school meals 0–1 times/week (as opposed to 2 times or more) were more frequently classified as having an unhealthy meal pattern.

**Table 2:** Schoolchildren estimates (proportion and 95% CI) by MESA levels in three years of WebCAAFE research

Variables	Scale Levels					
	**Healthy 18.3% (17.4–19.4)**		**Mixed 40.6% (39.0–42.3)**		**Unhealthy 41.0% (39.5–42.5)**	
**Survey year (*N* = 6,372)**	**%**	**95%CI**	**%**	**95%CI**	**%**	**95%CI**
2013	18.7	17.1–20.5	40.3	37.4–43.3	41.0	38.6–43.4
2014	18.3	16.8–19.8	40.9	37.9–44.0	40.8	37.7–44.0
2015	18.1	16.2–20.2	40.7	37.7–43.7	41.2	38.6–43.8
**Sex (*N* = 6,372)**						
Boys	17.7	16.2–19.4	38.9	36.8–41.1	43.3	41.5–45.2^d^
Girls	19.0	17.2–20.9	42.3	40.1–44.7	38.7	36.7–40.8^d^
**Age (*N* = 6,346)**						
7–9 years old	19.0	17.7–20.4	42.2	40.1–44.3	38.8	36.9–40.8^d^
10–12 years old	17.2	15.5–19.1	38.2	36.0–40.4	44.6	42.7–46.5^d^
**Family income (*N* = 6,372)**						
1º tertile	18.8	17.1–20.7	42.7	40.1–45.5	38.4	35.9–41.0^d^
2º tertile	18.2	16.3–20.2	40.6	37.6–43.7	41.2	38.9–43.6
3º tertile	17.9	16.0–19.9	37.7	35.2–40.3	44.4	41.6–47.2^d^
**School shift (*N* = 6,238)**						
Morning	19.2	18.2–20.3	39.0	36.6–41.5	41.8	39.6–44.1
Afternoon	17.5	15.8–19.4	42.3	40.1–44.5	40.2	38.1–42.3
**Weight status WHO**^a^** (*N* = 6,372)**						
Non-overweight, BMI Z-score for age < + 1	18.1	16.8–19.5	40.8	39.2–42.5	41.0	39.4–42.7
Overweight (including obesity), BMI Z-score for age ≥ + 1	18.8	17.2–20.4	40.3	37.2–43.3	41.1	38.5–43.6
**Day of food intake report (*N* = 6,372)**						
Non-school days	17.0	14.9–19.3	39.0	35.4–42.7	44.0	40.6–47.5
School days	18.8	17.7–20.0	41.2	39.5–42.9	40.0	38.6–41.4
**Frequency of school meal consumption (*N* = 5,462)**						
0–1 times/week	16.7	14.3–19.4	36.8	33.4–40.3^d^	46.5	43.5–49.5^d^
2–3 times/week	18.0	15.6–20.7	42.2	39.3–45.1	39.8	36.4–43.3^d^
4–5 times/week	19.1	17.5–20.8	44.6	42.2–47.0^d^	36.4	34.5–38.2^d^
**Physical Activity Score**^b^** (*N* = 6,372)**						
1º tertile	22.5	20.1–25.0^d^	33.5	30.8–36.3^d^	44.0	40.7–47.4^d^
2º tertile	18.6	17.1–20.3^d^	39.0	36.8–41.1^d^	42.4	40.2–44.6^d^
3º tertile	13.6	11.7–15.8^d^	50.1	46.9–53.3^d^	36.3	33.4–39.2^d^
** Screen activity**^**c**^**(*N*** **= 6,372)**						
1º tertile	22.0	20.5–23.6^d^	41.4	39.5–43.4	36.5	34.6–38.6^d^
2º tertile	18.6	16.5–20.9^d^	38.7	36.2–41.2	42.7	40.7–44.8^d^
3º tertile	11.7	10.1–13.5^d^	42.2	39.3–45.2	46.1	43.1–49.0^d^

*CI* Confidence Interval, *WebCAAFE* Food Intake and Physical Activities of Schoolchildren, *MESA* Meal and Snack Assessment Quality Scale, *N* total number of subjects

^a^Body mass index (BMI) was computed as weight (kg) divided by height squared (m). Age- and sex-specific BMI z-scores were calculated according to the World Health Organization [28]

^b^Physical activity score (PAS): created by multiplying the metabolic equivalent of each physical activity by the daily frequency reported [20]. The subject's PAS was the sum of all activity scores The first tertile was defined as the lowest, the second tertile as the intermediary, and the third tertile as the highest

^c^Daily frequency of sedentary behaviors based on screen activities (television, videogame, computer, tablet, cell phone). The first tertile was defined as the lowest, the second tertile as the intermediary, and the third tertile as the highest

^d^Statistical significance with the help of the confidence interval (non-overlapping confidence interval)

#### Characteristics of eating occasions

Most schoolchildren reported having lunch (96%), followed by dinner (90%) and breakfast (86%). Among snack times, afternoon snack was the most frequent (81%), followed by evening (63%) and morning (61%) snacks. Most schoolchildren (86%) consumed four or more meals/snacks per day.

At all snack times, there was a higher frequency of UPF consumption, whereas, at lunch and dinner, MPF consumption (rice, beans, and beef/poultry) was prevalent, although UPF was consumed in the form of sugary drinks (juices and sodas). The five most frequently reported WebCAAFE foods on each eating occasion, according to scale levels, are presented in Table 3. The healthy eating pattern was characterized by MPF consumption at all meals, particularly coffee with milk and fruit for breakfast; rice, beans, vegetables, and green leaves for lunch and dinner; beef/poultry for lunch; and fruits for snacks. The unhealthy pattern was characterized by UPF consumption, namely chocolate milk, cream cookies, yogurt, and breakfast cereal for breakfast; cream cookies, yogurt, chocolate milk, and fruit juices for morning and afternoon snacks; sodas and fruit juices at lunch and dinner; and sweets and pizza/ hamburger/hot dogs for the evening snack. The mixed pattern was characterized by simultaneous MPF and UPF consumption. In all meal patterns, bread consumption was observed on the majority of eating occasions.

**Table 3:** Top five foods per eating occasion by MESA Scale levels in three years of WebCAAFE research

Daily meal	Ranking	Healthy		Mixed		Unhealthy	
		**Food**	**%**	**Food**	**%**	**Food**	**%**
**Breakfast**	1	Bread	53	Bread	46	Bread	41
	2	Coffee with milk	46	Coffee with milk	30	Chocolate milk	35
	3	Milk	14	Chocolate milk	16	Cream cookies	15
	4	Fruit	11	Cream cookies, Milk	14	Yogurt	11
	5	Plain cake	7	Fruit	12	Breakfast cereal	10
**Morning Snack**	1	Fruit	18	Bread	20	Bread	13
	2	Bread	10	Fruit	16	Cream cookies	12
	3	Coffee with milk	6	Yogurt	12	Yogurt	10
	4	Yogurt, Plain cake, Porridge	4	Cream cookies	11	Fruit	6
	5	Milk	3	Chocolate milk, Fruit juices, Coffee with milk	8	Chocolate milk, Fruit juices	5
**Lunch**	1	Rice	59	Rice	56	Rice	56
	2	Beans	48	Beans	45	Beef/poultry	47
	3	Beef/poultry	43	Beef/poultry	43	Beans	41
	4	Green leaves, Vegetables, Pasta	13	Sodas, Fruit juices	16	Sodas	22
	5	Cassava flour	12	Green leaves	15	Fruit juices	16
**Afternoon Snack**	1	Bread	30	Bread	26	Bread	28
	2	Fruit	19	Fruit	16	Cream cookies	18
	3	Coffee with milk	18	Cream cookies	14	Chocolate milk	16
	4	Plain cake	11	Plain cake, Coffee with milk	13	Yogurt	13
	5	Cream cookies	6	Yogurt	12	Fruit juices	10
**Dinner**	1	Rice	36	Rice	34	Rice	33
	2	Beans	26	Beans	27	Beef/poultry	25
	3	Pasta, Vegetable soup	10	Beef/poultry	26	Beans	23
	4	Green leaves	8	Sodas	15	Sodas	19
	5	Vegetables, Cassava flour	7	Fruit juices	14	Fruit juices	15
**Evening Snack**	1	Fruit	12	Fruit	13	Sweets	12
	2	Bread	7	Fruit juices	9	Chocolate milk, Sodas	10
	3	Milk	6	Sweets, Sodas, Cream cookies	8	Fruit juices	8
	4	Coffee with milk	5	Chocolate milk, Bread, Yogurt, Plain cake	7	Bread, Yogurt	7
	5	Plain cake, Rice, Beans	4	Milk	6	Pizza/hamburger/hot dogs	6

*WebCAAFE* Food Intake and Physical Activities of Schoolchildren, *MESA* Meal and Snack Assessment Quality Scale

## Discussion

This study developed MESA, a unique scale to assess the quality of six daily meals according to the degree of food processing, using probabilistic IRT modeling to analyze dietary data from 6,399 schoolchildren obtained through the WebCAAFE questionnaire. The validity and reliability of the scale were evaluated, and the scale was applied to a representative sample of schoolchildren from southern Brazil. Previous studies focused mainly on analyzing overall diet quality or specific meals, mostly using CTT [3–5, 15, 16, 36].

MESA was shown to have adequate internal consistency and reliability according to IRT. Eleven out of 18 items exhibited good parameters, without DIF. MESA scores were categorized into three levels: healthy, mixed, and unhealthy. Higher scores represent greater consumption of UPF at daily meals. Most students in the sample had mixed (40.6%) and unhealthy (41%) meal patterns.

The GGUM model was applied, given that factor analysis showed the presence of a dominant factor explaining 27.4% of the data variance, reaching the assumption of unidimensionality. In the model, 11 items had adequate values of discrimination and location parameters, ranging from 0.70 to 1.37 and from − 0.88 to 0.75, respectively. The absence of extreme values confirms that the items are appropriate for measuring the construct of interest. Seven items had low discrimination and were not included. Discrimination is an important psychometric property for the item to differentiate individuals [33]. Five excluded items were related to PF consumption. Bread was the most reported PF. It should be noted that bread is the basis of the Brazilian diet, being one of the most consumed foods, according to the Brazilian National Dietary Survey [37]. Its consumption was similar among schoolchildren, regardless of the quality level of the meal, providing insufficient information for calibration. A sample of individuals with different consumption profiles would allow for a better fit of these items to the model.

After item positioning, it was possible to develop the MESA scale, describe the probability of consuming each type of food during meals and snacks, identify the quality patterns of meals in three levels, and evaluate the adequacy of meals to recommendations for a healthy diet. In MESA, higher scores represent greater participation of UPF in meals and snacks and, therefore, a worse meal quality.

The share of UPF in the diet can be used to measure diet quality [38], given that these foods have high energy density and unfavorable nutritional profile [39]. UPF consumption has been associated with adverse health outcomes [40], including increased body fat [41], serum lipids [42], and pro-inflammatory biomarker [43] in children and adolescents, reinforcing the importance of monitoring the dietary patterns of these population groups. The Dietary Guidelines for the Brazilian Population (DGBP) recommend that MPF and culinary preparations should be the basis of diets and that UPF consumption should be avoided. DGBP also provides additional recommendations on food choices and combination of foods in meals [18].

In our study, 41% of schoolchildren had an unhealthy meal pattern, contrary to dietary recommendations. UPF consumption during snack time, particularly morning snacks, was noticeable. The literature presents conflicting information regarding the influence of snacks on diet quality and energy balance. Snacking may lead to excessive energy intake and body weight gain, or contribute to adequate energy and nutrient intake [44]. The type of food and its quality influence total energy intake and the association between snacking and obesity [45]. In our study, the foods consumed as snacks are a potential focus for interventions. The concentration of schoolchildren near the item related to UPF consumption at morning snack time indicates the need for greater attention to the quality of foods consumed during this meal.

The scale proved to be suitable for the IRT model, confirming its internal consistency and reliability. The analyses conducted to assess the presence and nature of DIF, from the perspective of IRT, confirm the validity arguments that assume that the MESA items measure the same construct for all groups of interest, regardless of sex or age [21]. The TIC indicated that the instrument accurately measures meal quality scores between − 1.8 and 1.8. Unlike CTT, in which reliability can be estimated through an index, such as Cronbach's alpha, in IRT, the psychometric quality of an instrument can vary between latent trait levels, and this information is provided through TICs. Higher curves indicate higher psychometric quality, and the highest point represents the latent trait level at which the instrument provides more information. The idea that there is a single reliability for a given instrument is what differentiates CTT from IRT [46].

The current study has limitations. Despite NOVA having constraints regarding the accuracy of researchers in classifying foods by level of processing [47], it has been the most widely used method for evaluating diets according to food processing degree [48], being applied globally in epidemiological research to investigate the association between the consumption of UPF and diet quality and/or the potential effects of their consumption on human health [49]. Moreover, studies have demonstrated acceptable validity and reliability of dietary assessment tools to identify UPFF [50, 51]. The WebCAAFE was not designed to evaluate foods according to the NOVA system; therefore, some foods could be classified into more than one group. The most conservative or common classification was chosen. Porridge, plain cakes, vegetable soup, beans and mashed potatoes were considered prepared mixed dishes that were mainly based on unprocessed or minimally processed foods, for this reason were classified as MPF. This category includes all handmade dishes made from these foods and culinary ingredients such as fats, oils, salt, and sugar [17]. The decision-making process for classifying WebCAAFE foods according to their degree of processing was based on expert opinions. Regarding the foods consumed lacking sufficient information on preparation method or brand, the highest frequency of food consumption by the Brazilian population was used to estimate their level of processing. For example, given that the consumption frequency of yogurts and similar dairy beverages added with colorants and/or flavorings is higher than the natural yogurts, we opted to classify yogurt as UPF in this study [37]. Other limitations arise from the self-reported dietary assessment method, which is subject to underreporting [52]. Assessment of food intake was performed using a single 24-h recall, which may not be representative of the individual's usual intake, although it is widely accepted to assess food intake at a population level [52]. In addition, the day on which the WebCAAFE was applied differed between children. This strategy was used to describe the daily variability of dietary intake and physical activity over school days (Monday to Thursday) and non-school days (Sunday and holidays), allowing for analysis of these behaviors at the group level. Finally, the study population included children from public schools; it is unknown whether the results can be extrapolated to other populations, requiring further investigation.

Among the positive aspects of this study, it is worth mentioning that the IRT analyses were applied using a questionnaire validated for schoolchildren from two Brazilian municipalities [23, 26]. Another important contribution is that the scale was developed taking into account the three traditional Brazilian meals and snacks. WebCAAFE is structured according to six daily meals, with an objective definition of each occasion according to the time of day, reducing biases related to definitions of meals and snacks. Furthermore, the number of respondents was sufficient to obtain adequate estimates for item parameters, which showed low values for the standard error, indicating good precision. IRT opens new possibilities in the assessment of food consumption, and the created scale may be a new approach to assessing food consumption at different eating occasions according to the degree of food processing. The development of the scale was based on robust statistical analyses that complement the classical analysis, traditionally used in the development and validation of measuring instruments [33], as well as current recommendations for healthy eating. This is a pioneering study applying the unfolding IRT model to the assessment of food consumption during meals and snacks.

## Conclusions

IRT analysis allowed the development of the scale, which measures the quality of meals and snacks based on the degree of food processing. Once the item parameters are estimated, the values can be used to score any new response. The proposed scale can be applicable in multiple settings and population with the same precision, allowing for trend and longitudinal analysis, with the potential to assist in the monitoring, evaluation, and planning of healthy meals. The results can be used to track changes in dietary patterns and compare meal quality across different groups and populations.

Our results indicate the need for targeted nutritional interventions to improve the nutritional quality of snacks consumed by schoolchildren. Developing nutritional interventions by meal and targeting groups of individuals with similar meal quality levels seems promising, as it allows for greater action specificity. With information on meal patterns, it is possible to suggest better food choices for each eating occasion. For example, children with a healthy meal pattern should be encouraged to maintain healthy habits, such as consuming fruit as snacks. On the other hand, children with an unhealthy meal pattern should be encouraged to reduce the consumption of UPF (e.g., chocolate milk, cream cookies, and sugary drinks) and replace them with MPF. Future studies should apply the scale to other population groups and analyze its association with weight and socioeconomic status, demographic factors, and lifestyle characteristics.

### Supplementary Information

**Supplementary Material 1.**

**Supplementary Material 2.**

## Data Availability

The datasets used and/or analysed during the current study are available from the corresponding author on reasonable request.

## Abbreviations

BMI: Body Mass Index
CI: Confidence Interval
CTT: Classical Test Theory
DIF: Differential Item Functioning
GGUM: Generalized Graded Unfolding Model
ICC: Item Characteristic Curve
IRT: Item Response Theory
MESA: Meal and Snack Assessment Quality Scale
MPF: Unprocessed/Minimally Processed Foods
PAS: Physical Activity Score
PF: Processed Foods
SE: Standard Error
TIC: Test Information Curve
UPF: Ultra-Processed Foods
WebCAAFE: Food Intake and Physical Activities of Schoolchildren Questionnaire
