# Supplementary material for: Development and application of the Meal and Snack Assessment (MESA) quality scale for children and adolescents using item response theory
Source: Nutr J. 2024 May 14;23:50. doi: 10.1186/s12937-024-00948-y (PMC11092231; doi:10.1186/s12937-024-00948-y)
Supplement: Supplementary file 1 — Supplementary Material 1. [file 12937_2024_948_MOESM1_ESM.docx]

Additional file 1. Assessment of the differential item functioning (DIF) for sex (boys and girls) and age (7-9 and 10-12 years) of schoolchildren.

| **Sex** | **Scores**  **Intervals** | **Item** | | | | | | | | | | |
| --- | --- | --- | --- | --- | --- | --- | --- | --- | --- | --- | --- | --- |
|  |  | **i01** | **i03** | **i04** | **i05** | **i06** | **i09** | **i10** | **i12** | **i15** | **i16** | **i18** |
|  | 0 | -0.060 | 0 | -0.100 | 0.050 | 0 | 0 | -0.018 | -0.014 | 0 | -0.094 | 0 |
|  | 1 | 0.070 | -0.020 | -0.110 | -0.004 | -0.002 | 0.061 | -0.082 | -0.001 | -0.076 | -0.023 | 0.020 |
|  | 2 | -0010 | -0.090 | -0.030 | -0.010 | -0.007 | 0.006 | -0.079 | -0.109 | 0.007 | -0.100 | 0.060 |
|  | 3 | 0.020 | 0.020 | -0.020 | -0.022 | 0.094 | -0.051 | -0.087 | -0.051 | -0.043 | -0.002 | -0.015 |
|  | 4 | 0 | 0.010 | 0.010 | -0.042 | -0.086 | -0.036 | -0.032 | -0.042 | -0.041 | -0.061 | 0.036 |
|  | 5 | 0.020 | 0.040 | -0.020 | 0.016 | -0.020 | -0.051 | -0.039 | -0.063 | -0.032 | 0.017 | -0,072 |
|  | 6 | 0 | -0.020 | 0 | -0.038 | 0.019 | -0.063 | -0.012 | -0.067 | -0.052 | -0.006 | 0.009 |
|  | 7 | 0 | 0.040 | 0 | 0.008 | 0 | -0.043 | 0 | -0.049 | -0.033 | 0 | 0.039 |

*Cont.*

| **Age** | **Scores Intervals** | **Item** | | | | | | | | | | |
| --- | --- | --- | --- | --- | --- | --- | --- | --- | --- | --- | --- | --- |
|  |  | **i01** | **i03** | **i04** | **i05** | **i06** | **i09** | **i10** | **i12** | **i15** | **i16** | **i18** |
|  | 0 | -0.077 | 0 | 0.069 | -0.018 | 0 | 0 | -0.067 | -0.036 | 0 | 0.081 | 0 |
|  | 1 | 0.008 | 0.002 | 0.020 | 0.029 | 0.024 | 0.010 | -0.098 | -0.013 | -0.049 | -0.016 | 0,018 |
|  | 2 | 0.041 | 0.026 | 0.147 | -0.040 | 0.086 | 0.005 | -0.082 | -0.051 | 0.028 | 0.109 | 0.005 |
|  | 3 | 0.005 | 0.052 | 0.043 | -0.032 | 0.047 | -0.080 | 0.025 | -0.010 | -0.027 | 0.042 | 0.078 |
|  | 4 | 0.015 | 0.050 | 0.024 | 0.025 | -0.020 | -0.056 | -0.103 | 0.024 | -0.023 | 0.048 | -0.015 |
|  | 5 | 0.005 | -0.014 | -0.009 | 0.005 | 0.037 | -0.049 | -0.036 | -0.045 | -0.046 | -0.016 | 0.020 |
|  | 6 | 0 | -0.078 | 0.002 | -0.002 | -0.012 | -0.074 | -0.015 | -0.028 | -0.046 | -0.007 | -0.003 |
|  | 7 | 0 | -0,001 | 0 | -0.013 | 0 | -0.080 | 0 | -0.139 | -0.045 | 0 | 0.032 |

Note: (i01) MPF consumption at breakfast; (i03) UPF consumption at breakfast; (i04) MPF consumption at morning snack; (i05) PF consumption at morning snack; (i06) UPF consumption at morning snack; (i09) UPF consumption at lunch; (i10) MPF consumption at afternoon snack; (i12) UPF consumption at afternoon snack; (i15) UPF consumption at dinner; (i16) MPF consumption at evening snack; (i18) UPF consumption at evening snack.

MPF: unprocessed/minimally processed foods; PF: processed foods; UPF: ultra-processed food.
